# Reduction of in-hospital cardiac arrest with sequential deployment of rapid response team and medical emergency team to the emergency department and acute care wards

**DOI:** 10.1371/journal.pone.0241816

**Published:** 2020-12-01

**Authors:** Babith Mankidy, Christopher Howard, Christopher K. Morgan, Kartik A. Valluri, Bria Giacomino, Eddie Marfil, Prakruthi Voore, Yao Ababio, Javad Razjouyan, Aanand D. Naik, James P. Herlihy

**Affiliations:** 1 Department of Medicine, Baylor College of Medicine, Baylor St Luke’s Medical Center, Houston, Texas, United States of America; 2 Department of Medicine, Baylor College of Medicine, Houston, Texas, United States of America; 3 Veterans Affairs Health Services Research & Development, Center for Innovations in Quality, Effectiveness and Safety, Michael E. DeBakey VA Medical Center, Houston, Texas, United States of America; University of Hong Kong, HONG KONG

## Abstract

**Purpose:**

This study aimed to determine if sequential deployment of a nurse-led Rapid Response Team (RRT) and an intensivist-led Medical Emergency Team (MET) for critically ill patients in the Emergency Department (ED) and acute care wards improved hospital-wide cardiac arrest rates.

**Methods:**

In this single-center, retrospective observational cohort study, we compared the cardiac arrest rates per 1000 patient-days during two time periods. Our hospital instituted a nurse-led RRT in 2012 and added an intensivist-led MET in 2014. We compared the cardiac arrest rates during the nurse-led RRT period and the combined RRT-MET period. With the sequential approach, nurse-led RRT evaluated and managed rapid response calls in acute care wards and if required escalated care and co-managed with an intensivist-led MET. We specifically compared the rates of pulseless electrical activity (PEA) in the two periods. We also looked at the cardiac arrest rates in the ED as RRT-MET co-managed patients with the ED team.

**Results:**

Hospital-wide cardiac arrests decreased from 2.2 events per 1000 patient-days in the nurse-led RRT period to 0.8 events per 1000 patient-days in the combined RRT and MET period (p-value = 0.001). Hospital-wide PEA arrests and shockable rhythms both decreased significantly. PEA rhythms significantly decreased in acute care wards and the ED.

**Conclusion:**

Implementing an intensivist-led MET-RRT significantly decreased the overall cardiac arrest rate relative to the rate under a nurse-led RRT model. Additional MET capabilities and early initiation of advanced, time-sensitive therapies likely had the most impact.

## Introduction

The need for rapid response systems (RRS) evolved from reports in the 1990s showing that unexpected and possibly avoidable cardiac arrests were occurring too often in hospitalized patients [1, 2]. An estimated 60–85% of in-hospital cardiac arrests was prefaced by earlier clinical signals that indicated an increased risk of decompensation [3–5]. However, these are signals often missed or dismissed by clinicians [6–9]. Based on these reports, researchers hypothesized that deploying an early alert and rapid response team could potentially prevent cardiac arrests and improve patient outcomes [10].

In 2004, the Institute for Healthcare Improvement (IHI) began its “100,000 Lives Campaign,” which called for all United States hospitals to deploy rapid response teams [11]. Historically, these teams have had various configurations. The efferent limb of the rapid response system is a specialized intervention team that deploys to the patient’s bedside in response to physiologic derangements or clinical concerns of bedside nurses [12]. Different configurations of the RRS include a nurse-led Rapid Response Team (RRT) or a physician-led Medical Emergency Team (MET) [13, 14]. Although early literature used these interchangeably, recent reports have standardized the definitions of these teams [15]. For purposes of clarity in this study we define nurse-led team as RRT and intensivist-led team as MET.

Prior studies looking at the impacts of rapid response teams have shown mixed results. Some demonstrated a decrease in in-hospital mortality [16, 17] and hospital-wide cardiac arrest [18]. However, other reports failed to demonstrate a reduction in composite endpoints of death, unexpected cardiac arrest, and unplanned ICU admission [14, 19].

Our Institution adopted a nurse-led RRT in 2012 and after few years deployed an intensivist-led MET team in a sequentially deployed model. We sought to analyze the impact of this intervention on the cardiac arrest rates in the hospital. Our RRT and MET also co-managed complex patients who were admitted to the ICU but boarding in the emergency department (ED). There is limited data describing rapid response intervention systems in emergency departments [20].

We specifically looked at the impact of rapid response on pulseless electrical activity (PEA) arrest. From prior review of our hospital cardiac arrest data, PEA accounts for up to 70% of hospital cardiac arrests at our institution compared to published reports of 37% in the literature [21]. Older population, sepsis and increased burden of respiratory pathology are likely contributors of increased PEA [22]. We hypothesized that by adding an intensivist-led MET we would decrease the overall cardiac arrest rates and specifically PEA rates in the hospital by effectively managing conditions such as sepsis, severe hypoxia, and hypotension that lead to PEA [23].

## Methods

### Study setting

Baylor St. Luke’s Medical Center (BSLMC) is an 850-bed quaternary care teaching hospital in Houston, Texas, USA with a total of 120 ICU beds. In response to the IHI “100,000 Lives Campaign,” [11] BSLMC initiated a nurse-led RRT in 2013. Three experienced critical care nurses per shift staffed the RRT; during the shift, they assumed no other responsibilities outside of the RRT. In October 2014, the hospital initiated the intensivist-led MET, which had 24-hour staffing. The MET was staffed by a physician intensivist (24-hour coverage), an advanced nurse practitioner (12-hour daytime coverage), and a critical care fellow (18-hour daytime-evening coverage. The workflow for the RRT and RRT-MET was organized differently in the acute care wards and ED.

This study was submitted to the Baylor College of Medicine Institutional Review Board (H-38687) and determined not to constitute human subjects research.

### Acute care ward pathway

During the RRT period, the RRT carried a dedicated pager and phone, and nursing staff on the hospital wards could make calls to the RRT for patient evaluation at any time based on their clinical assessments. The RRT trigger criteria were internally developed based on established criteria of patient decompensation [24]. After receiving a call from nursing staff, the RRT then contacted the hospitalist, who subsequently determined if there was a need for ICU care. During the RRT-MET period, the RRT was activated by the same workflow, who then escalated to the 24-hour in-house MET. Based on acuity and need, the MET could initiate critical care interventions such as airway support, mechanical ventilation, vascular access, vasopressor initiation, and massive transfusion on the acute care ward. The RRT and MET continued to actively manage the patient on the ward until ICU transfer.

### ED pathway

RRT deployment to the ED only occurred in the second period. The MET was activated to respond to the ED specifically for patients in respiratory failure and shock. The ED physician could also call the MET for ad hoc conditions, such as severe acidosis or unstable airway, that might require advanced critical care expertise, or very frequent bedside assessments and interventions by an intensivist. The MET would typically call the RRT to provide nursing-level ICU support. The RRT nurses would, for example, set up pressure lines and titrate vasoactive medications. The MET goal was to assess all consults within 10–20 minutes of the initial activation. Patients in the ED who were determined to meet ICU admission criteria continued with MET management until transferred to the ICU.

### Data collection

Prospective data was collected for administrative operations of the rapid response service and entered into a database by the rapid response data manager. Cardiac arrest data were extracted and analyzed from the code sheets by a data abstractor on a weekly basis. To triangulate accuracy, the number of cardiac arrest alerts were correlated with the number of overhead code pages. Specific cardiac arrest rhythms were determined based on information from cardiac arrest sheets and notes. Cardiac arrest episodes were defined as complete cessation of circulation requiring cardio-pulmonary resuscitation (CPR). Demographic and hospital mortality data were extracted from the hospital electronic database system.

### Statistical analysis

Data were analyzed using SPSS (IBM, V25.0.0). The Fisher exact test was used to test the difference between categorical variables, and the t-test was used for the continuous variables for the pre-intervention and post intervention intervals, using a significance level of 0.05. To evaluate the effect of the implementation methods, we used logistic regression analysis (all the covariates fed to the model at the same time) on the outcomes of interest. The significance level, the odds ratio, and the 95% confidence interval estimated for the risk of parameters of interest were all reported when appropriate. To identify the possible moderation effect of demographics or clinical variables, we used the filter method. In the filter method, variables that met criteria (p-value less than 0.05) were chosen to be included as confounding factors to the regression model.

## Results

### Primary end point

In the RRT period, we analyzed 305,999 patient days between January 2013 and September 2014. In the RRT-MET group, we analyzed 550,617 patient days between October 2014 and December 2017. There was no statistical difference between the groups in terms of mean age or sex. There was a statistically significant increase in hospital length of stay (LOS), and Case Mix Index (CMI), and a non-statistically significant increase in mortality (**Table 1**). In the RRT period, there were 673 hospital-wide cardiac arrests, or 2.20 cardiac arrests per 1000 patient-days. In the RRT-MET period, there were 484 hospital-wide codes, or 0.88 cardiac arrests per 1000 patient-days. This event reduction translated to an odds ratio (OR) of 0.40 (0.35–0.45 95% CI) (p<0.001) (**Table 2**). More specifically, the cardiac arrest rates in the ICU reduced from 1.45 to 0.60 events per 1000 patient-days (p<0.001). In the acute care wards, the cardiac arrest rates reduced from 0.43 to 0.17 events per 1000 patient-days (p<0.001). In the ED, the cardiac arrest rate reduced from 0.20 to 0.07 events per 1000 patient-days (p<0.001).

**Table 1:** Baseline characteristics.

	Jan 2013—Sept 2014	Oct 2014—Dec 2017	
	RRT	RRT-MET	p-value
Total Patients (*n*)	44,643	77,898	
Female, (*n*) (%)	22,767 (51)	38,182 (49)	
Male, (*n*) (%)	21,875 (49)	39,716 (51)	
Age, years	61.5	61.7	0.309
Case Mix Index	2.048	2.394	<0.001
Length of Stay, Days	6.91	7.3	<0.001
Mortality Rate (%)	2.72	3.02	0.027

**Table 2:** Study outcomes before and after RRT-MET.

	RRT	RRT-MET	p-value	OR (95%CI)
	N (per 1000 patient days)	N (per 1000 patient days)		
Cohort Time	Jan 2013—Sept 2014	Oct 2014—Dec 2017		
Total Patient Days	305999	550617		
Cardiac Arrest Total	673 (2.20)	484 (0.88)	<0.001	0.40 (0.35, 0.45)
Total Pulseless Electrical Activity	474 (1.55)	273 (0.50)	<0.001	0.32 (0.28, 0.37)
Total Ventricular Fibrillation/ Tachycardia	147 (0.48)	112 (0.20)	<0.001	0.42 (0.33, 0.54)
Intensive Care Unit Cardiac Arrest	445 (1.45)	330 (0.60)	<0.001	0.41 (0.36, 0.47)
Intensive Care Unit Pulseless Electrical Activity	321 (1.05)	185 (0.34)	<0.001	0.32 (0.27, 0.38)
Acute Care Ward Cardiac Arrest	132 (0.43)	91 (0.17)	<0.001	0.38 (0.29, 0.50)
Acute Care Ward Pulseless Electrical Activity	78 (0.25)	52 (0.09)	<0.001	0.37 (0.26, 0.53)
Total Emergency Department Cardiac Arrest	60 (0.20)	41 (0.07)	<0.001	0.38 (0.26, 0.56)
Emergency Department Pulseless Electrical Activity	53 (0.17)	25 (0.05)	<0.001	0.26 (0.16, 0.42)

95%CI = 95 percent of confidence intervals.

### Secondary end points

**Fig 1:**
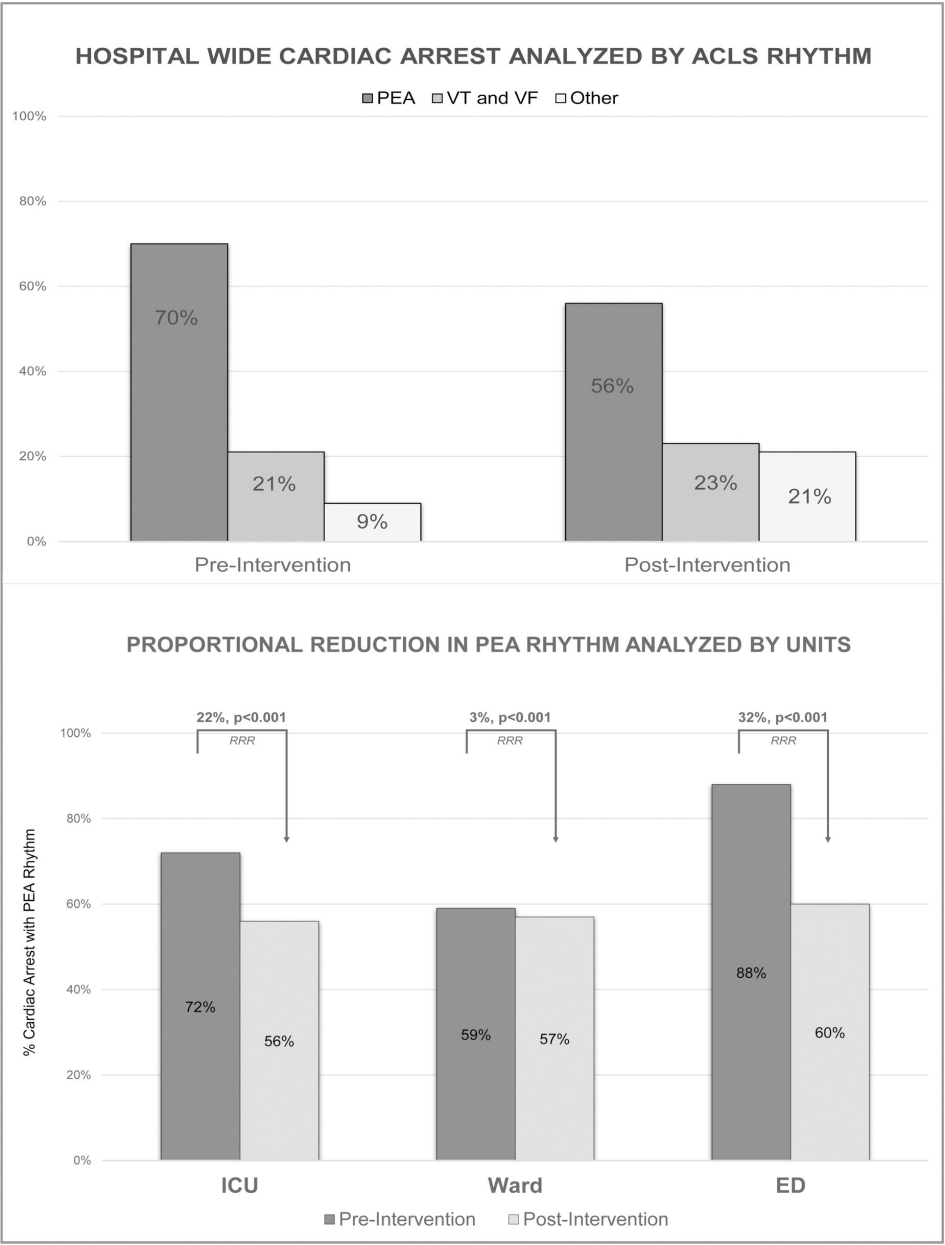
Cardiac arrest by ACLS and unit.

There was a statistically significant reduction in the number of overall in-hospital PEA arrests from 1.55 to 0.50 events per 1000 patient-days (p<0.001). PEA arrest contributed to 70% of ACLS rhythms in the RRT period compared to 56% in the RRT-MET period (**Fig 1A**). More specifically, there was a significant reduction in PEA arrests in all three sections of the hospital. ICU PEA arrest rates decreased from 1.05 to 0.34 per 1000 patient days (p<0.003), acute care ward PEA arrest rates decreased from 0.25 to 0.09 per 1000 patient days (p< 0.001), and ED PEA arrest rates decreased from 0.17 to 0.05 per 1000 patient days (p<0.003). The highest proportional drop in PEA arrest of 32% was noted in the ED (**Fig 1B)**. Total ventricular tachycardia/fibrillation (VT/VF) arrests decreased from 0.48 to 0.20 events per 1000 patient days (p<0.001) (**Table 2**).

**Fig 2:**
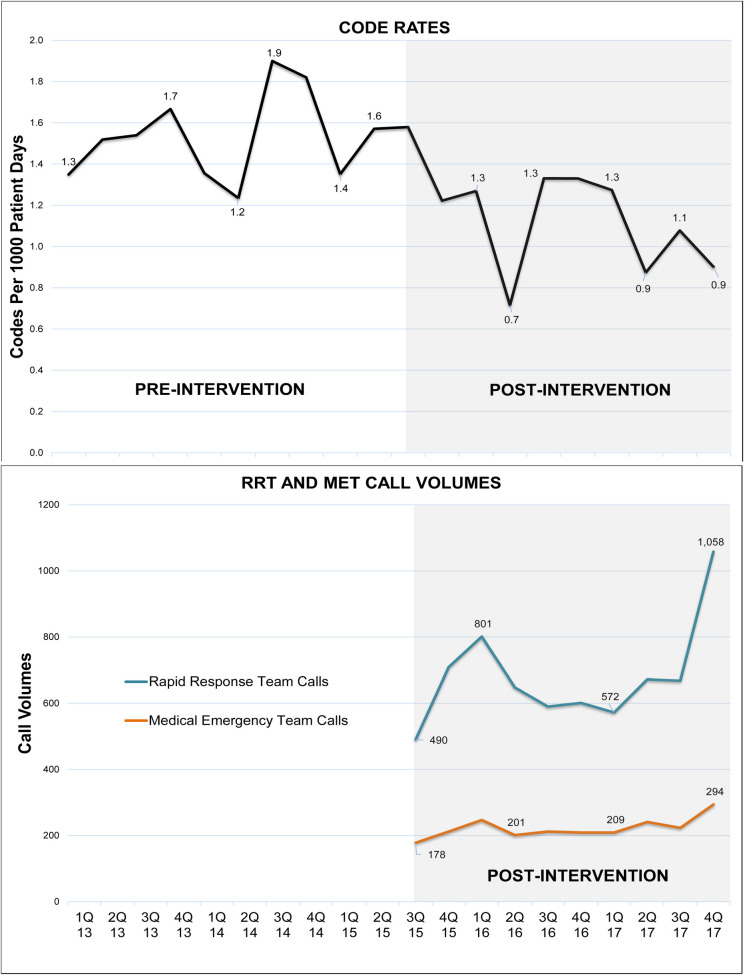
Code rate and RRT & MET call volume trends.

During the RRT-MET period, there was an incremental increase in utilization of RRT and MET over time. The median number of calls to the RRT was 658 calls per quarter, averaging to seven calls per day; the median number of calls to the MET was 212 calls per quarter, averaging to 2 calls per day. This correlated with a drop in overall code rates (**Fig 2**). The major reasons for ICU transfers following RRT and MET activation were respiratory instability (38%), cardiovascular instability (30%), and overall concern of the floor nursing staff.

## Discussion

The staffing model of the efferent arm of rapid response systems can vary widely and is largely dependent on the specific need of the hospital system and available resources [12, 25]. Failure to escalate properly is known to be one of the causes of failure to rescue in a rapid response event. Even after a patient is appropriately triaged, poor outcomes have been associated with delayed transfer to a higher level of care, delayed physician engagement, and of course delayed interventions [13]. A previous study looked at a comprehensive RRS that included an extensive educational program for clinicians on early recognition of shock, empowerment of front-line providers to use specific criteria to initiate therapy, protocol-driven and goal-directed therapy, and early transfer to the intensive care unit and its impact on overall and septic shock subgroup mortality. The results demonstrated that overall mortality decreased from 40% to 12%, while the septic shock subgroup mortality decreased from 50% to 10% [26]. Another study showed that each hour of delay in admission to the ICU was associated with a 1.5% increased risk of ICU death [27]. Outcomes improve with the addition of ICU-level care to rapid response services [28].

Our study demonstrates that the addition of an intensivist-led MET to the nurse-led RRT was associated with a significant reduction in the rates of hospital-wide cardiac arrest. Upon a deeper review of our results, several additional pertinent findings were identified which might help show where the impacts of the MET-RRT were most significant. First, the hospital location of the largest reduction in cardiac arrest rates was in the acute care wards, where the level of monitoring and response capacities are naturally the least intense. Second, our results indicate that a decrease in PEA arrests reduced overall cardiac arrests. PEA arrest contributed to 70% ACLS rhythms in the RRT period group compared to 56% in the RRT-MET period. A high proportional drop in PEA arrest was noted in the ED as well. PEA arrests most often result from associated with etiologies such as sepsis, hypoxia and hemorrhage [22]. RRT-MET interventions targeted at the timely management of these conditions may have helped reduce the PEA rates.

Reports on rapid response systems have focused primarily on their effects on the total number of arrests in the hospital, or their impact for arrests specifically on hospital wards. There has been some speculation that even a high functioning MET may simply divert an arrest from the ward to the ICU. Our data did not support that hypothesis. We were able to reduce the total cardiac arrest rates in all three main sections of the hospital: the ICU, ED, and acute care wards. We hypothesize that this was a result of early intervention in the ED and acute care wards. Several acute interventions such as intubation, central line placement, and activation of massive transfusion protocols occurred on both the acute care floor and ED while awaiting ICU beds. Avoiding delays in these and other critical care interventions while waiting for ICU bed improve outcomes [27, 29].

As ED workloads continue to increase, an emerging challenge for ED clinicians is how best to recognize and rapidly respond to deteriorating patients [30]. Additionally, it has been shown that delays in ICU admission from ED contribute to poor outcomes [31]. ED staffing resources vary widely, and hospitals such as ours that previously did not employ the ED-intensivist model find that dedicating time to managing critically ill patients is particularly challenging in a time-pressured environment. The first study of RRSs in the ED was published in 1995 [20]. Since then, the use of rapid response in the ED has gained popularity particularly in UK and Australia. In-hospital mortality rates are almost double in patients boarded in the ED for more than 2 hours compared with patients transferred within 2 hours of the admission order [32]. To this effect, our study showed benefit in cardiac arrest reduction by having RRT-MET co-manage complex cases that were admitted to ICU but delayed by ED throughput.

Another finding in our study was the correlation of the call volumes of RRT and MET with the decrease in cardiac arrest rates noted in other studies [33]. A dose response was demonstrated between in-hospital cardiac arrest and the amount of MET calls, with cardiac arrest rates reduced by 17% with an associated increase in MET calls, from 13.8 to 25.5 per 1000 admissions [34]. Increasing dosing of rapid response adds significant workload on physician-led teams and presents potential challenges for long-term sustainability. In our RRS staffing model, the nurse-led RRT was able to effectively triage ward calls, which helped maintain our afferent thresholds to sensitive levels without overloading the MET with large consult volumes. Even for a hospital of our size, with the use of nurse led RRT triage system, the MET calls averaged 2–3 calls per 12-hour shift.

## Study limitations

Our study has several limitations. First, with the retrospective nature of part of the study, we were unable to control for confounding factors affecting the cardiac arrest rates, such as changes in staffing and secular trends. The pre-post design without randomization limits the capacity to make causal inferences and significant claims on the external validity of our results. There is also perhaps the effect of having an intensivist see the patient in an outreach role that expedited goals of care discussions and drove the decrease in cardiac arrest [35]. Though it was included in the overall population of this study, there was not data to include asystole as a full subgroup for analysis. As with all single site, pre-post quality improvement studies, the presence of parallel quality improvement activities such as code blue improvement and hospital LOS improvement and staffing may serve as confounding variables.

## Conclusion

Although the literature review shows a variable impact of RRS on outcomes such as cardiac arrest and mortality, our study, with its unique staffing model, showed a significant decrease in overall code rates after the implementation of an intensivist-led MET in conjunction with nurse led RRT. The added capabilities of an ICU team to a nurse-led rapid response team and early initiation of advanced therapies likely had the highest impact on these reductions. We have found this model to be an efficient use of our MET physician resources in a high volume, academic, urban hospital setting, while seemingly retaining the expected effectiveness of a rapid response system. This may be a useful model for other hospital settings but requires further study.

## Future direction

Our experience suggests that the efferent limb of our RRS is adequate. Thus, as many investigators are currently doing, we would focus our efforts on investigating if an early warning system using electronic alert system could potentially engage our RRT and MET earlier and more reliably for decompensations, and hence change code rates in addition to overall hospital mortality. More recent research efforts have focused on the improvement in the ability of these systems to detect warning signs, minimize monitoring errors, and escalating care appropriately.

## Supporting information

S1 Dataset RRT-MET cardiac arrest dataset. (XLSX)
